# Physiological, Biochemical, and Transcriptomic Responses to Iron Deficiency in Two Potato Varieties

**DOI:** 10.3390/plants14182934

**Published:** 2025-09-21

**Authors:** Xiangying Ma, Yanping Zhang, Shenglong Yang, Miaomiao He, Yun Zhou, Guangji Ye, Jian Wang

**Affiliations:** Academy of Agriculture and Forestry Sciences, Qinghai University, Xining 810016, China; m18119349609@163.com (X.M.); qhyanping@163.com (Y.Z.); ysl890224@163.com (S.Y.); hemm0505@126.com (M.H.); zhouyun75@163.com (Y.Z.)

**Keywords:** potato, Fe deficiency, physiological and biochemical analysis, transcriptome

## Abstract

This study aimed to elucidate the physiological, biochemical, and transcriptional regulatory responses of potato plants to iron deficiency stress. Two potato varieties were selected for analysis: 05P (high tuber iron content) and CI5 (low tuber iron content). Tissue culture seedlings of both varieties were subjected to iron deficiency, and the effects on stem length, root length, fresh weight, soluble sugar and protein contents, as well as the activities of superoxide dismutase (SOD), peroxidase (POD), malondialdehyde (MDA), and leaf chlorophyll content (SPAD) values were evaluated. Additionally, the impact of iron deficiency on zinc (Zn), magnesium (Mg), calcium (Ca), manganese (Mn), and copper (Cu) concentrations in different tissues were analyzed. Transcriptomic sequencing and quantitative real-time PCR (qRT-PCR) were performed on various seedling tissues. The results showed that iron deficiency significantly inhibited seedling growth and development, resulting in reduced plant height and fresh weight, increased root length, decreased leaf SPAD content, and elevated soluble sugar and protein concentration. SOD, POD, and MDA activities were also significantly increased. Elemental analysis revealed that iron deficiency enhanced the uptake and accumulation of Zn, Mg, Ca, Mn, and Cu across different tissues. Transcriptomic analysis identified differentially expressed genes (DEGs) significantly enriched in pathways related to photosynthesis, carbon metabolism, and ribosome function in roots, stems, and leaves. Iron deficiency induced the upregulation of H^+^-ATPase genes in roots (PGSC0003DMG400004101, PGSC0003DMG400033034), acidifying the rhizosphere to increase active iron availability. Subsequently, this was followed by the upregulation of *FRO* genes (PGSC0003DMG400000184, PGSC0003DMG400010125, PGSC0003DMG401009494, PGSC0003DMG401018223), which reduce Fe^3+^ to Fe^2+^, and activation of *IRT* genes, facilitating Fe^2+^ transport to various tissues. Iron deficiency also reduced SPAD content in leaves, negatively impacting photosynthesis and overall plant growth. In response, the osmotic regulation and antioxidant defense systems were activated, enabling the plant to mitigate iron deficiency stress. Additionally, the absorption and accumulation of other metal ions were enhanced, likely as a compensatory mechanism for iron scarcity. At the transcriptional level, iron deficiency induced the expression of genes involved in metal absorption and transport, as well as those related to photosynthesis, carbon metabolism, and ribosomal function, thereby supporting iron homeostasis and maintaining metabolic balance under stress conditions.

## 1. Introduction

Iron reversibly cycles between Fe^2+^ and Fe^3+^ through redox reactions. As a key component of the plant electron transport chain and various coenzymes, it plays a vital role in numerous physiological and metabolic processes, including photosynthesis, respiration, nitrogen fixation, amino acid synthesis, and reactive oxygen species (ROS) scavenging [1,2,3,4,5]. In neutral and alkaline soils, iron predominantly exists as Fe^3+^, which has extremely low solubility. As a result, the available iron concentration in such soils often falls below the 10^−9^–10^−4^ M range required for normal plant growth and development [6,7]. Consequently, iron deficiency limits the growth of many plant species. Photosynthetic organisms are particularly sensitive to fluctuations in iron availability. Under iron-deficient conditions, photosynthetic efficiency declines sharply, ferritin levels are altered, and symptoms such as leaf chlorosis and disrupted chloroplast ultrastructure emerge [8,9,10]. To adapt to iron-limited environments, plants have evolved distinct iron acquisition mechanisms, with plants being classified as Mechanism I plants and Mechanism II plants [11]. Dicotyledonous plants, such as potatoes, and non-graminaceous monocots employ Mechanism I, which involves enhanced iron solubilization and uptake. In response to iron deficiency, they induce the expression of genes encoding plasma membrane-localized Fe^3+^-chelate reductase (*FRO*) and Fe^2+^ transporters (*IRT*) in root epidermal cells, while also secreting low-molecular-weight compounds such as carboxylic acids, flavins, and phenolics to mobilize iron from the soil [12,13]. In contrast, graminaceous monocots, such as maize, use Mechanism II, which relies on the secretion of phytosiderophores—iron-chelating compounds that bind Fe^3+^ and facilitate its uptake via specific transporters [14].

Under iron-deficient conditions, the expression of the *FRO* gene in plants increases 4 –5 fold compared to normal growth conditions, enabling more efficient iron utilization from the soil [15]. The *IRT1* gene, a member of the *ZIP* family, is essential for transporting Fe^2+^ from the roots to various tissues in *Arabidopsis thaliana*. *IRT1* mutant plants exhibit impaired iron uptake, leading to severe chlorosis, stunted growth, and, in some cases, seedling lethality [16,17]. Several transcription factors are critical for maintaining iron homeostasis in plants. FIT has been identified as a central transcription factor that regulates iron uptake in Arabidopsis roots by modulating the expression of 448 genes, including *FRO2* and *IRT1* [18]. Since the first *bHLH* transcription factor involved in iron uptake was identified in tomato, subsequent studies have shown that the homologous gene *AtbHLH29* (also known as *FER*) in Arabidopsis plays a key role in regulating *FRO2* and *IRT1* expression [19,20,21,22]. Additionally, members of the *MYB* transcription factor family, such as *MYB72* and *MYB10*, have been found to contribute to plant tolerance to iron deficiency in alkaline soils [23].

Over one-third of the world’s farmland comprise calcareous soils, which often induce iron deficiency in crops. Studies have shown that peanuts grown on such soils exhibit reduced dry weight in leaves, stems, and roots, leading to a 20% yield loss. In other crops—including trees, grains, vegetables, and legumes—yield losses may exceed 25% [24]. The potato (*Solanum tuberosum* L.), native to the Andes Mountains, is the fourth most important food crop after rice, wheat, and corn [25,26]. It is widely cultivated worldwide due to its high yield, adaptability, and rich content of easily absorbable nutrients. Micronutrients such as iron, manganese, and zinc are essential for the early growth and development of potato plants and play key roles in tuber yield and quality formation [27]. Deficiencies in these elements can cause substantial reductions in tuber yield and nutritional value [28,29]. Genotypic differences in tuber iron content have been reported in potatoes [30,31]. To investigate the effects of iron deficiency stress at the physiological, biochemical, and transcriptomic levels, we conducted experiments using two potato varieties: the high-tuber-iron variety 05P and the low-tuber-iron variety CI5. We characterized the phenotypic, physiological, and biochemical responses of these varieties to iron deficiency and identified candidate genes and pathways potentially involved in the potato’s iron response. Therefore, these findings enhance our understanding of the molecular mechanisms underlying iron homeostasis in potatoes and provide a theoretical basis for future iron biofortification efforts.

## 2. Materials and Methods

### 2.1. Plant Materials

The high tuber iron-content variety 05P (tuber iron content: 66.36 mg/kg DW) and the low tuber iron-content variety CI5 (tuber iron content: 1.16 mg/kg DW) were selected from 345 potato germplasm resources in our laboratory. Virus-free tissue culture seedlings of 05P and CI5 were preserved in the germplasm resource library at the Institute of Biotechnology, Qinghai University. Virus-free test-tube seedlings retrieved from the library were subcultured for three generations on 1/2 solid MS medium. Two-week-old seedlings were then cut into ~1.5 cm stem segments, each containing 1–2 growth points (lateral meristem), and cultured on experimental 1/2 MS solid medium.

After 10 days of growth (with 4–5 leaves), plants with uniform development were selected. The roots were rinsed three times with ultrapure water and then transferred to 1/2 MS solid medium with iron stress (FeNaEDTA concentration: 1 mg/L for iron deficiency stress vs. 40.4 mg/L for the control), according to the method described by Legay et al. [13]. The EDTA content in the iron-stress medium was adjusted to 40.4 mg/L using Na_2_EDTA, and the pH of all media was adjusted to 5.8 with 1 M NaOH, and 25 g/L of sugar and 7 g/L of agar were added. The specifications of the heat-resistant PC tissue culture bottles used are as follows: capacity 440 mL, upper opening diameter 55 mm, bottom diameter 80 mm, height 110 mm. Plants were cultivated in an artificial climate chamber at 20 °C under a 16 h light/8 h dark photoperiod (LED light source was used, set at 100 μmol·m^−2^·s^−1^, with standardized timing set at 08:00–24:00), with 85–95% relative humidity. Tissue samples were collected after 7 or 14 days for various physicochemical measurements, transcriptome sequencing, and qRT-PCR analysis. All media and ultrapure water were sterilized by autoclaving prior to use. Expansion and transplantation of tissue culture seedlings were performed in a laminar flow hood. All reagents, including ½ MS medium, FeNaEDTA, Na_2_EDTA, and NaOH, were purchased from Sangon Biotech (Shanghai, China).

### 2.2. Measurement of Morphological Indicators

After exposing two varieties of test-tube seedlings to different 1/2MS iron-deficient solid medium for seven days, stem length, root length, and biomass were measured as follows:

Stem length: The distance from the base of the stem to the growth point of the tissue culture seedling was measured in 10 plants per treatment, and the average value was calculated (unit: cm).

Root length: The length from the root–stem junction to the tip of the longest root was measured in 10 plants per treatment, and the average value was recorded (unit: cm).

Fresh shoot weight: The fresh shoot weight of the entire shoot (stem and leaves) was measured in 10 plants per treatment, and the average value was calculated (unit: g).

### 2.3. Measurement of Physiological Indicators

At 7 and 14 days of iron deficiency stress, complete leaves were sampled from the test-tube seedlings of both varieties for physiological analysis. Soluble sugar content, soluble protein content, SOD activity, POD activity, MDA content, and SPAD values were determined using reagent kits A145-1-1, A045-2, A001-1, A084-3-1, A003-1, and A147-1-1, respectively, all from Nanjing Jiancheng Biotechnology Research Institute (Nanjing, China). Measurements were performed according to the instructions provided with each kit.

### 2.4. Determination of Metal Element Content

After seven days of growth on the stress medium, entire plantlets of both varieties were harvested, rinsed five times with deionized water, dried, and then separated into roots, stems, and leaves. The samples were dried at 80 °C in a constant-temperature ventilated oven until a constant weight was reached. The dried tissues were ground into a fine powder, and 0.1 g of each tissue was weighed. Each sample was digested overnight in a 50 mL conical flask using 5 mL of HNO_3_:H_2_O_2_ (3:1, *v*/*v*). Digestion was completed at 150 °C on a ceramic electric heating plate until a clear, colorless solution was obtained. The digest was transferred to a 25 mL volumetric flask and brought to volume with 5% HNO_3_. The Fe, Zn, Cu, Ca, Mg, and Mn concentrations were determined using flame atomic absorption spectrometry (novAA400P, Analytik Jena, Germany). Each sample was analyzed in triplicate. All chemical reagents used were of analytical grade. Glassware was soaked overnight in 20% HNO_3_, rinsed with ultrapure water, dried, and used only after complete preparation. HNO_3_ and H_2_O_2_ were purchased from Sangon Biotech (Shanghai, China). Standard stock solutions of Fe, Zn, Cu, Ca, Mg and Mn were obtained from the National Analysis Center for Iron and Steel (NCS, Beijing, China).

### 2.5. Illumina Sequencing, RNA Extraction, Reverse Transcription, qRT-PCR Analysis

For 7-day-old tissue culture seedlings, samples were collected from roots, stems, and leaves, each with three biological replicates, and placed in 5 mL capped centrifuge tubes. The samples were immediately frozen in liquid nitrogen and stored at −80 °C for RNA-Seq (performed by Avison Biotechnology, Beijing, China) and qRT-PCR analysis (sample information is provided in Table 1). Total RNA was isolated from all samples using the PureLink™ RNA Mini Kit (Invitrogen, Waltham, MA, USA), followed by genomic DNA removal with PureLink™ DNase (Invitrogen, USA). To ensure high purity, the RNA was further cleaned up using the RNeasy Mini Kit (Qiagen, Hilden, NRW, Germany). RNA quality was assessed by measuring absorbance ratios (A260/A280 = 1.8–2.0 and A260/A230 ≥ 2.0) with a NanoDrop spectrophotometer (Thermo Fisher Scientific, Waltham, MA, USA), and integrity was verified using an Agilent 2100 Bioanalyzer (Agilent Technologies, Santa Clara, CA, USA). For cDNA library construction, poly(A)+ mRNA was enriched using oligo(dT) magnetic beads and fragmented. First-strand cDNA was synthesized with random hexamer primers, followed by second-strand cDNA synthesis. The double-stranded cDNA was purified using AMPure XP beads (Beckman Coulter, Brea, CA, USA) and amplified by PCR to construct sequencing libraries. High-quality libraries were defined as those with an effective concentration >4 nM. Finally, all 36 cDNA libraries were subjected to paired-end sequencing (PE150) on an Illumina HiSeq 2500/4000 platform (Illumina, San Diego, CA, USA) at Beijing Allwegene Technology Co., Ltd. (Beijing, China). Raw sequencing data were subjected to stringent quality control using the following criteria: (1) removal of reads containing sequencing adapters; (2) elimination of reads with >10% ambiguous bases (N); and (3) exclusion of reads with >50% of bases exhibiting low quality scores (Q ≤ 20). The resulting high-quality clean reads were then aligned to the reference genome (PGSC_DM_v6.1; http://solanaceae.plantbiology.msu.edu/pgsc_download.shtml, accessed on 18 February 2023) using STAR (Spliced Transcripts Alignment to a Reference; version 2.7.10a) with default parameters. Transcriptome assembly was performed using Cufflinks (version 2.1.1; http://cole-trapnell-lab.github.io/cufflinks, accessed on 20 February 2023) to identify novel transcripts from the mapped RNA-Seq reads. Differential alternative splicing events were analyzed using rMATS (http://rnaseq-mats.sourceforge.net/index.html, accessed on 20 February 2023) with default settings. The raw sequencing data generated in this study have been deposited in the NCBI Sequence Read Archive (SRA) under the accession number: PRJNA1312044.

Based on RNA-seq analysis, differentially expressed genes (DEGs) associated with iron absorption and transport were selected for experimental validation. Gene-specific primers were designed using Primer Premier 5 (Premier Biosoft, San Francisco, CA, USA) based on the coding sequences (CDS) of target DEGs, and subsequently synthesized by Sangon Biotech (Shanghai, China; https://www.sangon.com/). Total RNA was extracted from different tissues of potato plantlets (two cultivars under various treatments) using the MiniBEST Universal RNA Extraction Kit (TaKaRa, Kusatsu, Shiga, Japan). RNA quality was assessed by 1.2% agarose gel electrophoresis and spectrophotometric analysis (NanoDrop, Wilmington, DE, USA). Samples with A260/A280 ratios between 1.8–2.1 were considered high-quality and used for subsequent experiments. First-strand cDNA was synthesized using the PrimeScript™ RT reagent Kit (TaKaRa, Kusatsu, Shiga, Japan) according to the manufacturer’s protocol. Quantitative real-time PCR (qPCR) was performed using TB Green^®^ Premix Ex Taq™ II (TaKaRa, Kusatsu, Shiga, Japan) on a CFX96 Real-Time PCR System (Bio-Rad, Hercules, CA, USA). Each 20 μL reaction contained: 10 μL TB Green Premix Ex Taq II, 0.4 μL each of forward and reverse primers (10 μM), 2 μL cDNA template (corresponds to approximately 100 ng of RNA), and 7.2 μL nuclease-free water. The thermal cycling conditions consisted of an initial denaturation at 95 °C for 30 s, followed by 40 cycles of 95 °C for 10 s and 60 °C for 32 s. Three biological replicates were analyzed for each sample, with three technical replicates per biological replicate. Cycle threshold (Ct) values were collected using Bio-Rad CFX Maestro™ Software (version 2.3, Bio-Rad, Hercules, CA, USA). Relative gene expression levels were calculated using the 2^−ΔΔCt^ method method [32], with reference genes listed in Table 2 for normalization.

### 2.6. Identification of Differentially Expressed Genes

Gene expression under iron deficiency stress was quantified using HTSeq2 (https://www.illumina.com.cn/systems/sequencing-platforms.html, accessed on 25 November 2022). Subsequently, the FPKM (Fragments Per Kilobase of transcript per Million mapped fragments) values of each gene were calculated based on the gene length, followed by obtaining their count data, and finally mapped to the corresponding genes. FPKM accounts for sequencing depth and gene length, and is a widely used metric for quantifying gene expression [33]. Differential expression analysis was performed in R (v3.3.2) using the three biological replicates from the RNA-Seq experiment. Genes with an adjusted *p*-value < 0.05 were considered differentially expressed.

### 2.7. Analysis of the Functions of Differentially Expressed Genes

To analyze the functions of differentially expressed genes, the GOseqR package [34] and KOBAS 2.0 [35] were used. Gene Ontology (GO) and Kyoto Encyclopedia of Genes and Genomes (KEGG) enrichment analyses were performed for the differentially expressed genes. Adjusted *p*-values < 0.05 were considered indicative of significant enrichment [36]. GO enrichment analysis was used to study the functional classification of differentially expressed genes, while KEGG pathway analysis identified significantly enriched pathways.

### 2.8. Statistical Analysis

Data were summarized and organized using Excel 2016, and normality tests, variance analysis, and post-hoc comparison plots were conducted using Origin 2021. Data were analyzed using one-way ANOVA followed by Fisher’s Least Significant Difference (LSD) test for post hoc comparisons when ANOVA indicated significant differences (*p* < 0.05).

## 3. Result

### 3.1. The Effect of Iron Deficiency Stress on the Growth of Potato Tissue Culture Seedlings

**Figure 1:**
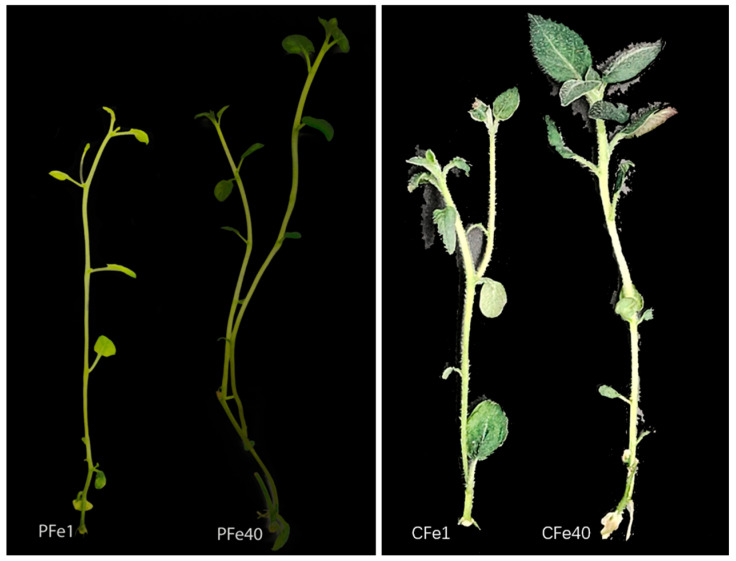
Growth status of tissue culture seedlings under iron deficiency stress at 14 days. PFe1 (iron-deficient treatment of potato cultivar 05P, 1 mg/L FeNaEDTA), PFe40 (control for 05P with normal iron supply, 40.4 mg/L FeNaEDTA), CFe1 (iron-deficient treatment of cultivar CI5, 1 mg/L FeNaEDTA), and CFe40 (control for CI5 with normal iron supply, 40 mg/L FeNaEDTA).

Figure 1 illustrates that after 14 days of treatment with different concentrations of FeNaEDTA, the growth and morphology of test-tube seedlings from two potato varieties exhibited marked differences compared to the control group. The 05P seedlings were more sensitive to iron deficiency stress: their young leaves displayed pronounced chlorosis, and both stem and leaf development were restricted. Compared to the control, their stems were significantly thinner, and the leaves were fewer and smaller. In the CI5 group, iron deficiency also significantly inhibited growth, although chlorosis of the young leaves was less apparent. Overall, stem and leaf development in CI5 was visibly poorer than in the control group.

### 3.2. The Influence of Iron Deficiency Stress on the Morphological Indicators of Potato Tissue Culture Seedlings

#### 3.2.1. The Effect of Iron Deficiency Stress on the Height of Potato Tissue Culture Seedlings

**Figure 2:**
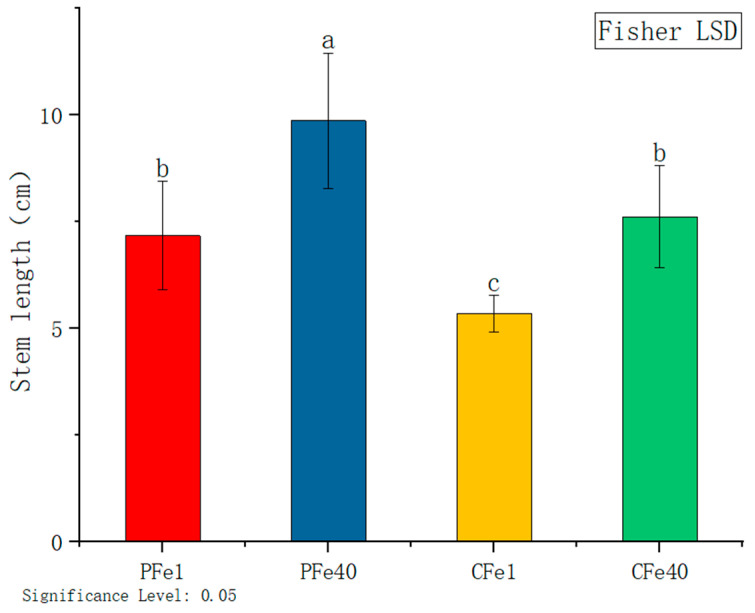
Stem length of different varieties under iron deficiency stress. Different letters above error bars indicate values are significantly different at the 0.05 level according to Fisher’s LSD test. Sample information is detailed in Table 1.

In Figure 2, the effects of iron deficiency on plant height are evident after 7 days of FeNaEDTA treatment. The plant height of 05P was significantly reduced by 27.3%, while that of CI5 significantly decreased by 29.8%, relative to their respective controls. These results demonstrate a significant inhibitory effect of iron deficiency on the plant height of both varieties.

#### 3.2.2. The Effect of Iron Deficiency Stress on the Root Length of Potato Tissue Culture Seedlings

**Figure 3:**
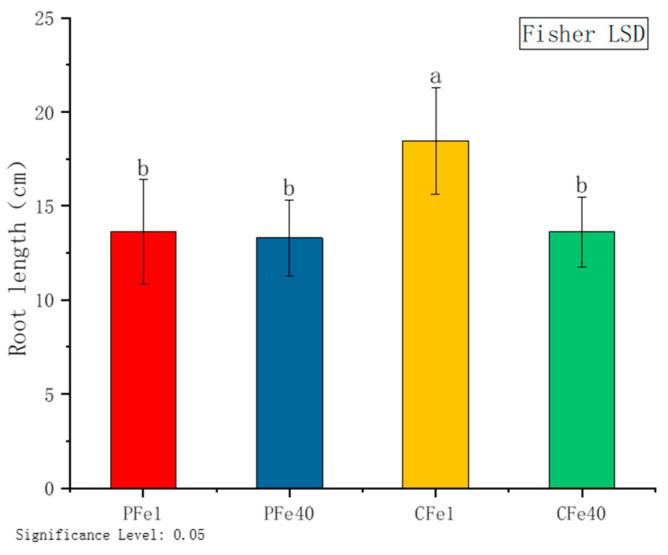
Root length of different cultivars under iron deficiency stress. Different letters above error bars indicate values are significantly different at the 0.05 level according to Fisher’s LSD test. Sample information is detailed in Table 1.

As illustrated in Figure 3, following the exposure of test-tube seedlings of two potato varieties to varying concentrations of FeNaEDTA stress for 7 days, the root length of 05P under iron deficiency stress was observed to be 2.4% longer than that of the control group, with no statistically significant difference. In contrast, the root length of CI5 under iron deficiency stress increased by 35.7% compared to the control group, which was found to be statistically significant.

#### 3.2.3. The Effect of Iron Stress on the Fresh Shoot Weight of Potato Tissue Culture Seedlings

**Figure 4:**
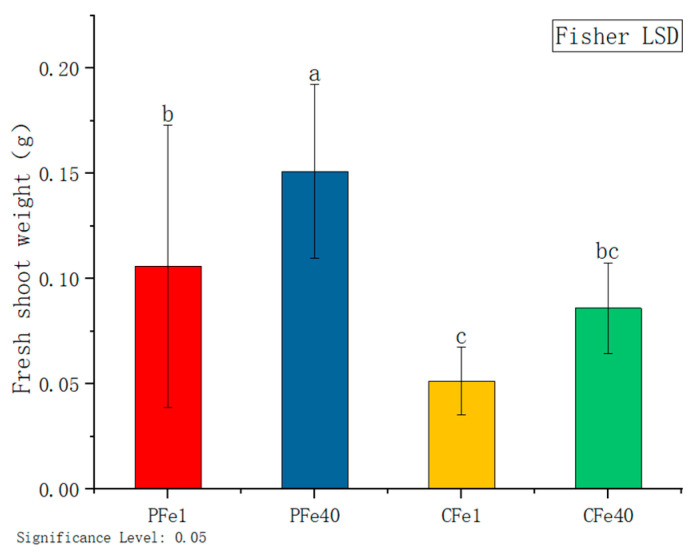
Fresh shoot weight of different plant varieties under iron deficiency stress. Different letters above error bars indicate values are significantly different at the 0.05 level according to Fisher’s LSD test. Sample information is detailed in Table 1.

Figure 4 illustrates that after seven days of iron deficiency stress, the test-tube seedlings of both potato varieties exhibited significant reductions in fresh shoot weight. Specifically, the fresh shoot weight of the 05P variety was 29.8% lower than that of the control group, while the CI5 variety showed a 40.4% decrease compared to its control. These findings indicate that iron deficiency had a significant impact on the seedling fresh shootweight of both varieties.

### 3.3. The Influence of Iron Deficiency Stress on the Physiological Indicators of Potato Tissue Culture Seedlings

#### 3.3.1. The Effect of Iron Deficiency Stress on the Soluble Sugar Content of Potato Tissue Culture Seedlings

**Figure 5:**
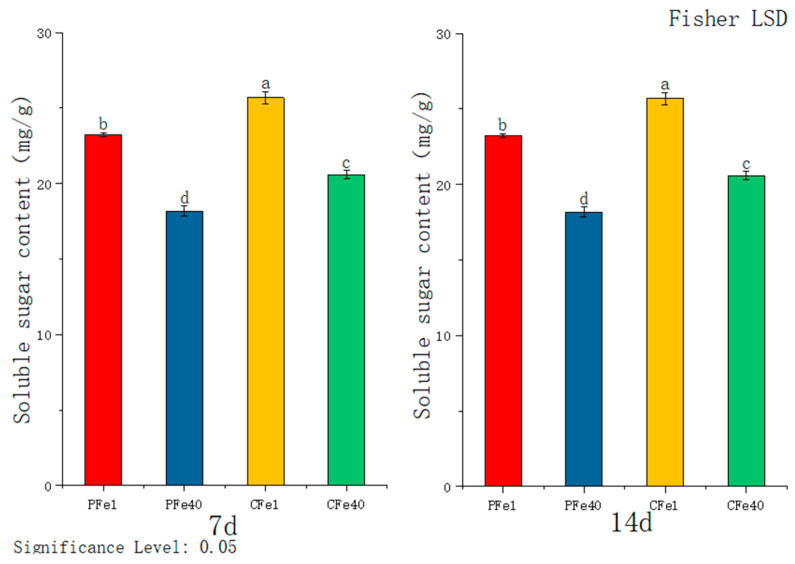
Soluble sugar content in different plant varieties under iron deficiency stress (7 days/14 days). Different letters above error bars indicate values are significantly different at the 0.05 level according to Fisher’s LSD test. Sample information is detailed in Table 1.

According to Figure 5, the soluble sugar content in the leaves of both potato varieties increased significantly after 7 and 14 days of iron deficiency stress compared to their respective controls. At 7 days, the sugar content in 05P leaves was significantly higher (27.8%) than in the control group, and although still significantly elevated at 14 days, it declined slightly over time. In the CI5 variety, the soluble sugar content was significantly higher (24.83%) than the control at 7 days and 19.55% higher at 14 days. Compared to the 7-day time point, soluble sugar levels decreased in both the stress and control groups as the duration of stress increased. Nevertheless, under iron deficiency stress, the tissue culture seedlings of both 05P and CI5 consistently exhibited significantly higher soluble sugar levels than their respective controls.

#### 3.3.2. The Effect of Iron Deficiency Stress on the Soluble Protein Content of Potato Tissue Culture Seedlings

**Figure 6:**
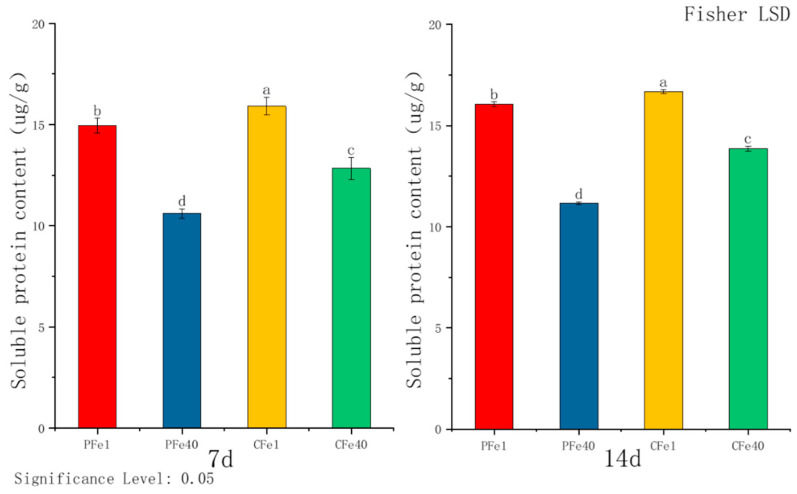
Soluble protein content in different plant varieties under iron deficiency stress (7 days/14 days). Different letters above error bars indicate values are significantly different at the 0.05 level according to Fisher’s LSD test. Sample information is detailed in Table 1.

Figure 6 illustrates that after 7 days of iron deficiency stress, the soluble protein content in the leaves of 05P plants increased significantly by 40.9% compared to the control group, while CI5 leaves significantly increased by 23.9%. The increase was more pronounced in 05P leaves under iron deficiency stress. By day 14, the soluble protein content in 05P leaves rose by 43.81%, and in CI5 leaves by 20.43%, relative to their respective controls. As stress duration increased, soluble protein levels continued to rise in leaves of both varieties, with 05P consistently showing a greater increase.

#### 3.3.3. The Effect of Iron Deficiency Stress on the Superoxide Dismutase (SOD) Activity of Potato Tissue Culture Seedlings

**Figure 7:**
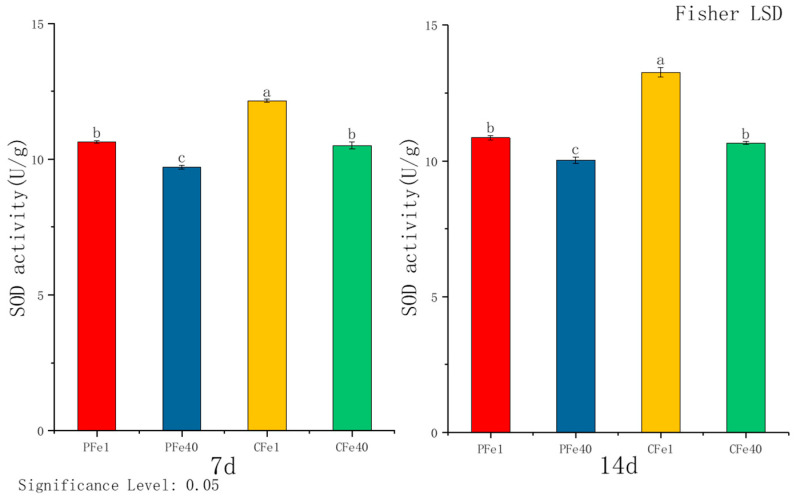
Changes in SOD activity in different cultivars under iron deficiency stress (7 days/14 days). Different letters above error bars indicate values are significantly different at the 0.05 level according to Fisher’s LSD test. Sample information is detailed in Table 1.

According to Figure 7, after 7 days of iron deficiency stress, SOD activity in 05P leaves increased by 9.58% and in CI5 leaves by 15.7% relative to controls. By day 14, SOD activity in 05P and CI5 leaves was elevated by 8.21% and 24.48%, respectively. In both varieties, SOD activity increased progressively with the duration of iron deficiency stress.

#### 3.3.4. The Effect of Iron Deficiency Stress on the Peroxidase (POD) Activity of Potato Tissue Culture Seedlings

**Figure 8:**
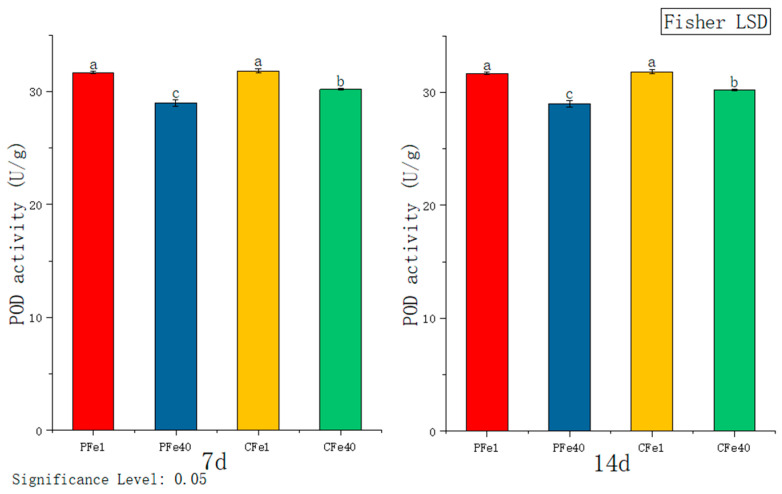
Changes in POD activity in different plant varieties under iron deficiency stress (7 days/14 days). Different letters above error bars indicate values are significantly different at the 0.05 level according to Fisher’s LSD test. Sample information is detailed in Table 1.

Figure 8 illustrates that after 7 days of iron deficiency stress, the POD activity in the leaves of 05P and CI5 tissue culture seedlings increased significantly by 8.97% and 5.44%, respectively, compared to their corresponding controls. After 14 daysof stress, POD activity in 05P and CI5 leaves increased by 6.57% and 6.99%%, respectively, relative to the control. With prolonged stress duration, POD activity continued to rise in CI5 leaves.

#### 3.3.5. The Effect of Iron Deficiency Stress on the Malondialdehyde (MDA) Activity of Potato Tissue Culture Seedlings

**Figure 9:**
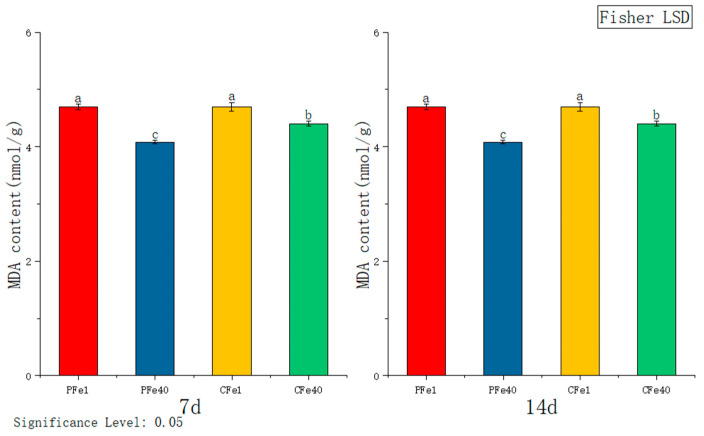
Changes in MDA content in different cultivars under iron deficiency stress (7 days/14 days). Different letters above error bars indicate values are significantly different at the 0.05 level according to Fisher’s LSD test. Sample information is detailed in Table 1.

According to Figure 9, after 7 days of iron deficiency stress, MDA levels in 05P and CI5 leaves increased significantly by 35.73% and 33.23%, respectively, compared to the controls. At 14 days, MDA levels in 05P and CI5 leaves remained significantly elevated, by 4.85% and 4.83%, respectively.

#### 3.3.6. The Effect of Iron Deficiency Stress on the SPAD Content of Chlorophyll in Potato Tissue Culture Seedlings

**Figure 10:**
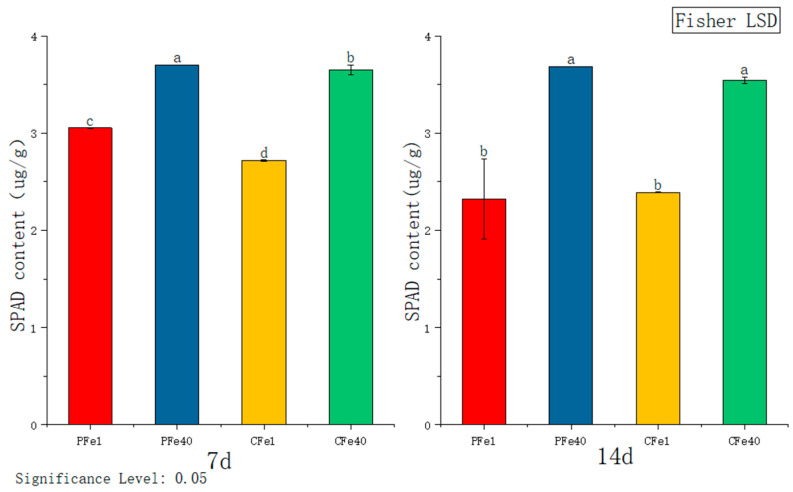
Changes in SPAD content in different plant varieties under iron deficiency stress (7 days/14 days). Different letters above error bars indicate values are significantly different at the 0.05 level according to Fisher’s LSD test. Sample information is detailed in Table 1.

Figure 10 illustrates that after 7 days of iron deficiency stress, the SPAD content in the leaves of 05P and CI5 tissue culture seedlings decreased significantly compared to their respective controls—by 17.47% and 25.57%, respectively. After 14 days of stress, the SPAD content in 05P and CI5 leaves was significantly lower by 36.94% and 32.61%, respectively, relative to the control. With increasing stress duration, SPAD content continued to decline in both 05P and CI5 leaves.

### 3.4. The Effect of Iron Deficiency Stress on Metal Elements in Different Tissues of Potato Tissue Culture Seedlings

**Figure 11:**
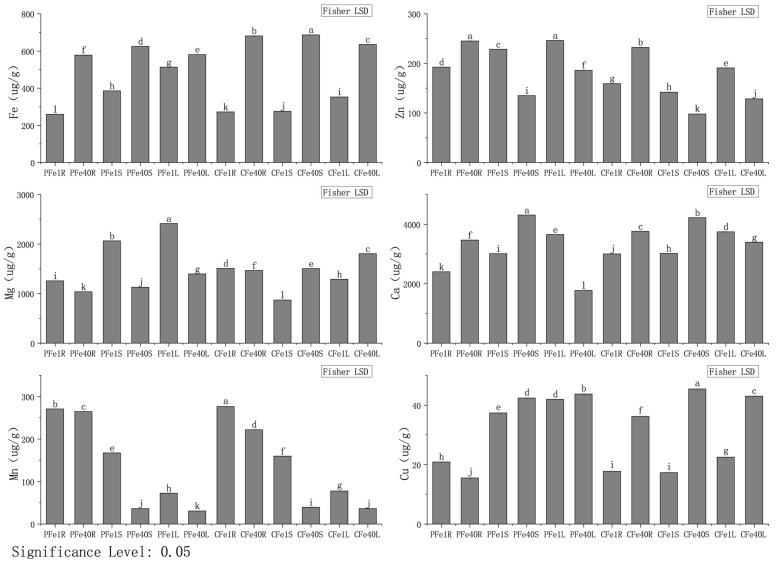
Metal element contents in different tissues of potato tissue culture seedlings under iron deficiency stress (7 days). Different letters above error bars indicate values are significantly different at the 0.05 level according to Fisher’s LSD test. PFe1R/S/L represents the root (R), stem (S), and leaf (L) tissues of genotype 05P under iron-deficient conditions (1 mg/L FeNaEDTA), while PFe40R/S/L denotes the corresponding tissues of 05P under control (40.4 mg/L FeNaEDTA). Similarly, CFe1R/S/L and CFe40R/S/L refer to the root, stem, and leaf tissues of CI5 under iron-deficient (1 mg/L FeNaEDTA) and control (40.4 mg/L FeNaEDTA) conditions, respectively. Sample information is detailed in Table 1.

As shown in Figure 11, after 7 days of iron deficiency stress, the Fe content in the roots of the 05P group decreased significantly by 7.35% compared to the control group, while the Zn, Mg, Ca, Mn, and Cu contents increased significantly by 186.63%, 21.42%, 44.27%, 71.32%, and 34.06%, respectively. In the stems of the 05P group, the Fe content decreased significantly by 6.35%, whereas the Zn, Mg, Ca, Mn, and Cu contents increased significantly by 70.18%, 82.56%, 108.99%, 59.05%, and 19.89%, respectively, compared to the control. In the leaves, the Fe content decreased significantly by 26.51%, whilethe Zn, Mg, Ca, Mn, and Cu contents increased significantly by 33.18%, 72.79%, 162.75%, 135.25%, and 155.2%, respectively. Among the roots, Mn showed the greatest increase under iron deficiency stress, followed by Cu. In contrast, Fe content in the stems decreased significantly by 51.32%, while Zn, Mg, Ca, Mn, and Cu contents increased significantly. In the leaves, Fe content decreased significantly by 44.43%, while Zn, Mg, Ca, Mn, and Cu contents increased significantly by 40.78%, 39.78%, 59.87%, 1227.99%, and 161.02%, respectively, compared to the control group.

### 3.5. Identification of Differentially Expressed Genes in Different Tissues of 05P and CI5 Under Iron Deficiency Stress

**Figure 12:**
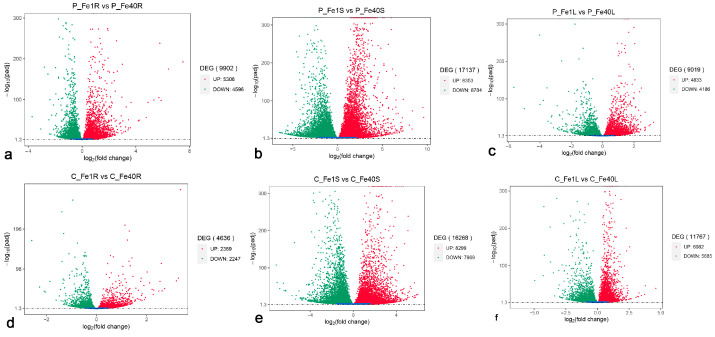
Differentially expressed genes (DEGs) in different tissues of 05P and CI5 under iron deficiency stress. Note: The horizontal and vertical axes represent the fold change in gene expression and the statistical significance of differential expression, respectively. Red dots indicate significantly upregulated DEGs, green dots indicate significantly downregulated DEGs, and blue dots represent non-significant DEGs. Sample information is detailed in Table 1. (**a**) DEGs in 05P roots (**b**) DEGs in 05P stems. (**c**) DEGs in 05P leaves.(**d**) DEGs in CI5 roots. (**e**) DEGs in CI5 stems. (**f**) DEGs in CI5 leaves.

The statistical results of DEGs in various tissues of 05P and CI5 under iron deficiency stress are shown in Figure 12. The DEG analysis for different tissues of 05P is presented in Figure 12a–c. In the roots of 05P (P_Fe1RvsP_Fe40R), a total of 9902 DEGs were identified, including 5306 up-regulated and 4596 down-regulated genes. In the stems (P_Fe1SvsP_Fe40S), 17,137 DEGs were identified, comprising 8353 up-regulated and 8784 down-regulated genes. In the leaves (P_Fe1LvsP_Fe40L), 9019 DEGs were detected, of which 4833 were up-regulated and 4186 were down-regulated. The corresponding analysis for CI5 is shown in Figure 12d–f. In the roots of CI5 (C_Fe1RvsC_Fe40R), 4636 DEGs were identified, including 2389 up-regulated and 2247 down-regulated genes. In the stems (C_Fe1SvsC_Fe40S), 16,268 DEGs were identified, comprising 8299 up-regulated and 7969 down-regulated genes. In the leaves (C_Fe1LvsC_Fe40L), 11,767 DEGs were detected, of which 6082 were up-regulated and 5685 were down-regulated.

### 3.6. Differential Expression Gene GO Enrichment Analysis

**Figure 13:**
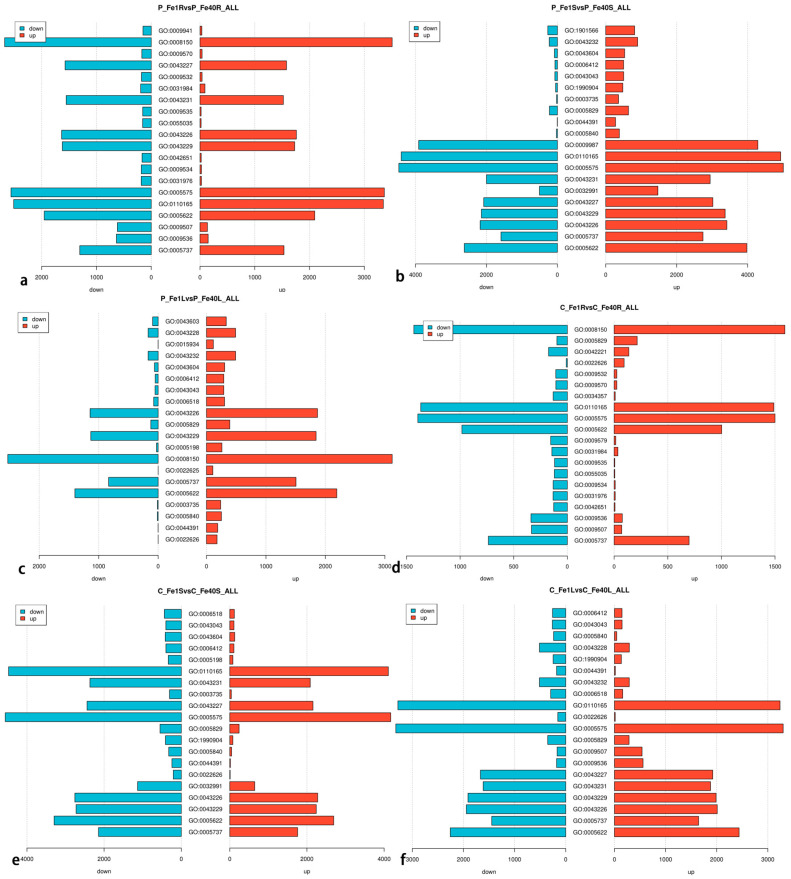
GO enrichment analysis of DEGs in different tissues of 05P and CI5 under iron deficiency stress. Sample information is detailed in Table 1. (**a**) GO enrichment analysis of DEGs in 05P roots. (**b**) GO enrichment analysis of DEGs in 05P stems.(**c**) GO enrichment analysis of DEGs in 05P leaves. (**d**) GO enrichment analysis of DEGs in CI5 roots. (**e**) GO enrichment analysis of DEGs in CI5 stems. (**f**) GO enrichment analysis of DEGs in CI5 leaves.

The results of GO enrichment analysis for DEGs are shown in Figure 13. DEGs detected in 05P roots were analyzed for GO enrichment, and the results are presented in Figure 13a. Among the top 20 significantly enriched GO categories in 05P roots (P_Fe1RvsP_Fe40R), one belonged to the biological_process category and 19 to the cellular_component category. In the biological_process category, DEGs were significantly enriched in biological_process (GO:0008150). In the cellular_component category, significant enrichment was observed in cytoplasm (GO:0005737), plastid (GO:0009536), chloroplast (GO:0009507), intracellular (GO:0005622), cellular anatomical entity (GO:0110165), cellular_component (GO:0005575), plastid thylakoid (GO:0031976), chloroplast thylakoid (GO:0009534), thylakoid membrane (GO:0042651), and intracellular organelle (GO:0043229). DEGs detected in 05P stems (P_Fe1SvsP_Fe40S) were also subjected to GO enrichment analysis, with results shown in Figure 13b. The top 20 significantly enriched GO terms included five in the biological_process category, 14 in the cellular_component category, and one in the molecular_function category. In the biological_process category, DEGs were significantly enriched in organonitrogen compound biosynthetic process (GO:1901566), peptide biosynthetic process (GO:0043043), translation (GO:0006412), amide biosynthetic process (GO:0043604), and cellular process (GO:0009987). In the cellular_component category, enrichment was observed in intracellular (GO:0005622), cytoplasm (GO:0005737), organelle (GO:0043226), intracellular organelle (GO:0043229), membrane-bounded organelle (GO:0043227), protein-containing complex (GO:0032991), intracellular membrane-bounded organelle (GO:0043231), cellular component (GO:0005575), and cellular anatomical entity (GO:0110165). In the molecular*_function* category, DEGs were significantly enriched in structural component of ribosome (GO:0003735). Similarly, DEGs detected in 05P leaves (P_Fe1LvsP_Fe40L) were analyzed for GO enrichment, and the results are shown in Figure 13c. Among the 20 most significantly enriched GO terms, six belonged to the biological_process category, 12 to the cellular_component category, and two to the molecular_function category. In the biological_process category, DEGs were significantly enriched in translation (GO:0006412), peptide biosynthetic process (GO:0043043), amide biosynthetic process (GO:0043604), and cellular amide metabolic process (GO:0043603). In the cellular_component category, significant enrichment was observed in organelle (GO:0043226), intracellular organelle (GO:0043229), cytoplasm (GO:0005737), non-membrane-bounded organelle (GO:0043228), and intracellular non-membrane-bounded organelle (GO:0043232). In the molecular_function category, DEGs were significantly enriched in structural constituent of ribosome (GO:0003735) and structural molecule activity (GO:0005198).

The DEGs identified in CI5 roots(C_Fe1RvsC_Fe40R) were subjected to GO enrichment analysis, and the results are presented in Figure 13d. Among the 20 most significantly enriched GO categories, 2 belonged to the biological_process category and 18 to the cellular_component category. Within the biological_process category, DEGs were significantly enriched in biological_process (GO:0008150) and response to chemical (GO:0042221). Within the cellular_component category, significant enrichment was observed in cytoplasm (GO:0005737), chloroplast (GO:0009507), plastid (GO:0009536), thylakoid membrane (GO:0042651), plastid thylakoid (GO:0031976), chloroplast thylakoid (GO:0009534), plastid thylakoid membrane (GO:0055035), chloroplast thylakoid membrane (GO:0009535), and organelle subcompartment (GO:0031984). Similarly, the DEGs identified in CI5 stems(C_Fe1SvsC_Fe40S) were analyzed for GO enrichment, and the results are shown in Figure 13e. Among the 20 most significantly enriched GO categories, 4 were from the biological_process category, 14 from the cellular_component category, and 2 from the molecular_function category. In the biological_process category, the DEGs in CI5 stems under iron deficiency stress were significantly enriched in translation (GO:0006412), amide biosynthetic process (GO:0043604), peptide biosynthetic process (GO:0043043), and peptide metabolic process (GO:0006518). In the cellular_component category, the enriched GO terms included cytoplasm (GO:0005737), intracellular (GO:0005622), intracellular organelle (GO:0043229), organelle (GO:0043226), protein-containing complex (GO:0032991), cytosolic ribosome (GO:0022626), ribosomal subunit (GO:0044391), ribosome (GO:0005840), ribonucleoprotein complex (GO:1990904), and cytosol (GO:0005829). In the molecular_function category, DEGs were significantly enriched in structural constituent of ribosome (GO:0003735) and structural molecule activity (GO:0005198).

The DEGs detected in the CI5 leaves (C_Fe1LvsC_Fe40L) under iron deficiency stress were subjected to GO enrichment analysis. The results are shown in Figure 13f. It was found that among the 20 most significantly enriched GO categories of DEGs in the CI5 leaves, 3 belonged to the biological_process category and 17 to the cellular_component category. In the biological_process category, DEGs were significantly enriched in GO terms such as translation (GO:0006412), peptide biosynthetic process (GO:0043043), and peptide metabolic process (GO:0006518); in the cellular_component category, DEGs were significantly enriched in GO terms such as intracellular (GO:0005622), cytoplasm (GO:0005737), organelle (GO:0043226), intracellular organelle (GO:0043229), intracellular membrane-bounded organelle (GO:0043231), membrane-bounded organelle (GO:0043227), plastid (GO:0009536), chloroplast (GO:0009507), and cytosol (GO:0005829).

### 3.7. KEGG Enrichment Analysis of Differentially Expressed Genes in Potatoes Under Iron Deficiency Stress

**Figure 14:**
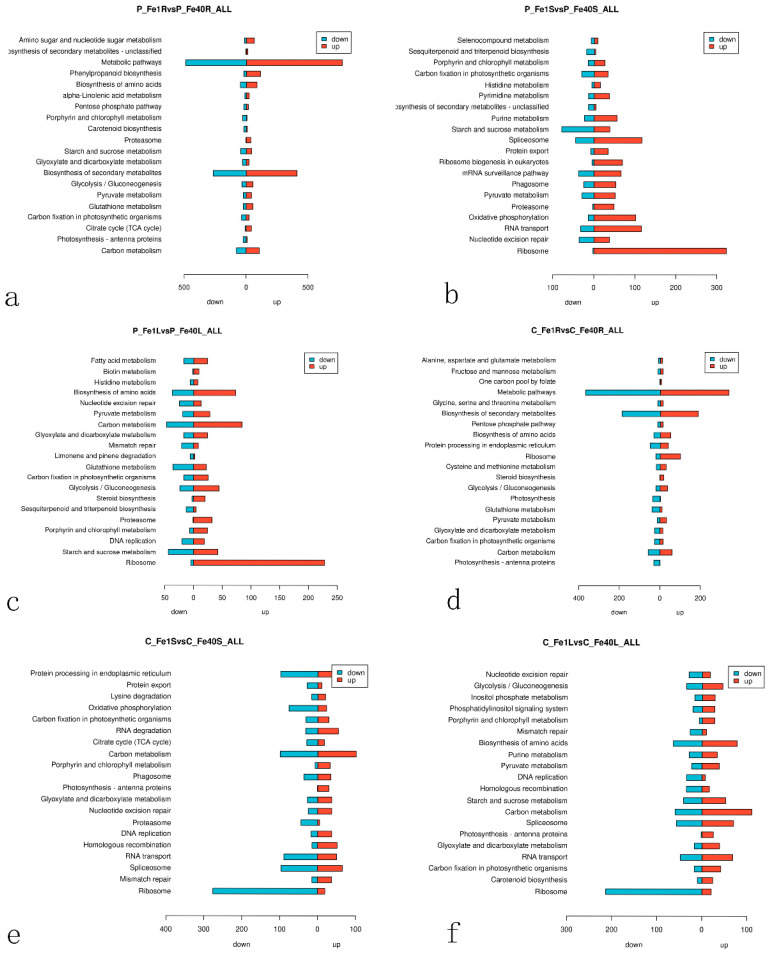
KEGG enrichment analysis of DEGs in different tissues of 05P and CI5 under iron deficiency stress. Sample information is detailed in Table 1. (**a**) KEGG enrichment analysis of DEGs in 05P roots. (**b**) KEGG enrichment analysis of DEGs in 05P stems. (**c**) KEGG enrichment analysis of DEGs in 05P leaves. (**d**) KEGG enrichment analysis of DEGs in CI5 roots. (**e**) KEGG enrichment analysis of DEGs in CI5 stems. (**f**) KEGG enrichment analysis of DEGs in CI5 leaves.

In living organisms, different genes coordinate to perform various biological functions. Pathway significance enrichment analysis helps identify the most critical biochemical metabolic and signal transduction pathways involving DEGs [37]. The KEGG enrichment analysis of DEGs in different tissues of 05P and CI5 under iron deficiency stress is shown in Figure 14. Figure 14a presents the KEGG enrichment analysis of DEGs in the 05P root (P_Fe1RvsP_Fe40R), with significantly enriched pathways including Carbon metabolism (sot01200), Photosynthesis-antenna proteins (sot00196), Citrate cycle (sot00020), Carbon fixation in photosynthetic organisms (sot00710), Glutathione metabolism (sot00480), Pyruvate metabolism–Glycolysis (sot00620), and Gluconeogenesis (sot00010), among others. In these pathways, most DEGs are upregulated. Figure 14b shows the KEGG enrichment analysis of DEGs in the 05P stem (P_Fe1SvsP_Fe40S). Significantly enriched pathways include Ribosome (sot03010), Nucleotide excision repair (sot03420), RNA transport (sot03013), Oxidative phosphorylation (sot00190), and Proteasome (sot03050). Here, gene upregulation is predominant, especially in the ribosome pathway, where nearly all DEGs are upregulated. Figure 14c illustrates the KEGG enrichment analysis of DEGs in the 05P leaf (P_Fe1LvsP_Fe40L). The significantly enriched pathways include Ribosome (sot03010), Starch and sucrose metabolism (sot00500), DNA replication (sot03030), Porphyrin and chlorophyll metabolism (sot00860), and Proteasome (sot03050). In each of these pathways, the majority of DEGs are upregulated.

The KEGG pathway enrichment analysis of DEGs in CI5 roots (C_Fe1RvsC_Fe40R) is shown in Figure 14d. The significantly enriched pathways include Photosynthesis-antenna proteins (sot00196), Carbon metabolism (sot01200), Carbon fixation in photosynthetic organisms (sot00710), Photosynthesis (sot00630), and Glycolysis/Gluconeogenesis (sot00620). Among these, many DEGs are down-regulated, indicating that iron deficiency affects various metabolic processes in potato roots. The KEGG pathway enrichment analysis of DEGs in CI5 stems (C_Fe1SvsC_Fe40S) is shown in Figure 14e. Significantly enriched pathways include Ribosome (sot03010), Mismatch repair (sot03430), Spliceosome (sot03040), RNA transport (sot03013), Homologous recombination (sot03440), and DNA replication (sot03030). In CI5 leaves (C_Fe1LvsC_Fe40L) (Figure 14f), significantly enriched pathways include Ribosome (sot03010), Carotenoid biosynthesis (sot00906), Carbon fixation in photosynthetic organisms (sot00710), RNA transport (sot03013), Glyoxylate and dicarboxylate metabolism (sot00630), Photosynthesis-antenna proteins (sot00196), Spliceosome (sot03040), Carbon metabolism (sot01200), and Starch and sucrose metabolism (sot00500).

### 3.8. Potato Root, Stem and Leaf DEGs Related to Fe

Overall, the GO and KEGG enrichment analyses revealed that the regulatory networks significantly enriched by DEGs in potato test-tube seedlings under iron deficiency stress involve pathways related to photosynthesis, carbon metabolism, ribosomes, and others. As the primary objective was to understand the differential regulatory networks governing iron and other divalent metal cation homeostasis in various potato tissues under iron deficiency stress, we focused on DEGs associated with the transport of these metal ions.

Based on the significantly enriched pathways identified through GO and KEGG enrichment analyses, a total of 137 DEGs belonging to various gene families were screened and found to be associated with metal absorption and transport, particularly iron. Among these, 82 DEGs were expressed in 05P roots, with 43 upregulated and 39 downregulated, while 55 DEGs were expressed in CI5 roots, with 33 upregulated and 22 downregulated. Forty-six DEGs exhibited the same expression pattern in the roots of both varieties—28 upregulated and 18 downregulated (Supplementary S1). In the stems, 107 DEGs were detected in 05P (44 upregulated and 63 downregulated), and 99 in CI5 (44 upregulated and 55 downregulated). Nineteen DEGs showed the same expression pattern in the stems of both varieties, with 5 upregulated and 14 downregulated (Supplementary S1). In the leaves, 70 DEGs were identified in 05P (43 upregulated and 27 downregulated), and 80 in CI5 (43 upregulated and 37 downregulated). Twenty-seven DEGs had the same expression pattern in the leaves of both varieties—13 upregulated and 14 downregulated (Supplementary S1).

**Figure 15:**
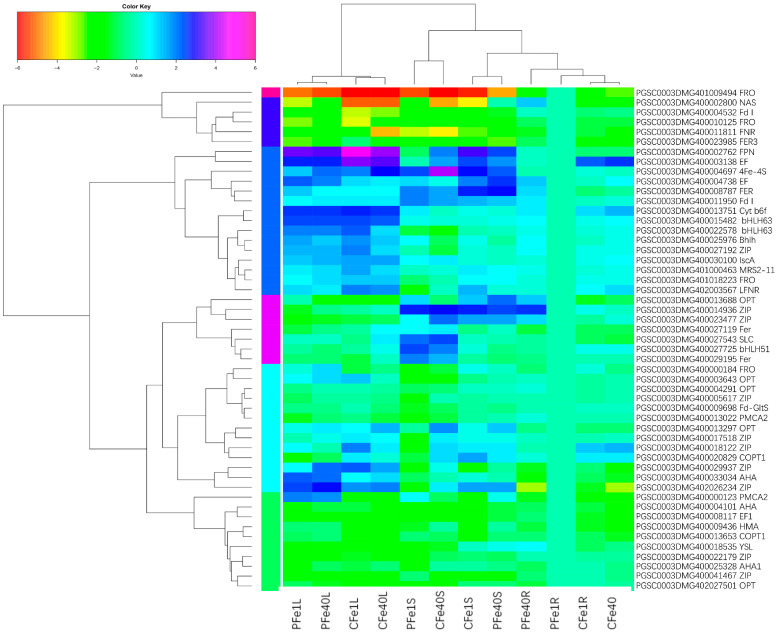
Heatmap and hierarchical clustering analysis of DEGs differentially expressed in at least four out of six comparisons (P_Fe1RvsP_Fe40R, P_Fe1SvsP_Fe40S, P_Fe1LvsP_Fe40L_ALL, C_Fe1RvsC_Fe40R, C_Fe1SvsC_Fe40S, C_Fe1LvsC_Fe40L) in 05P and CI5 under Fe-deficient and control conditions.

**Figure 16:**
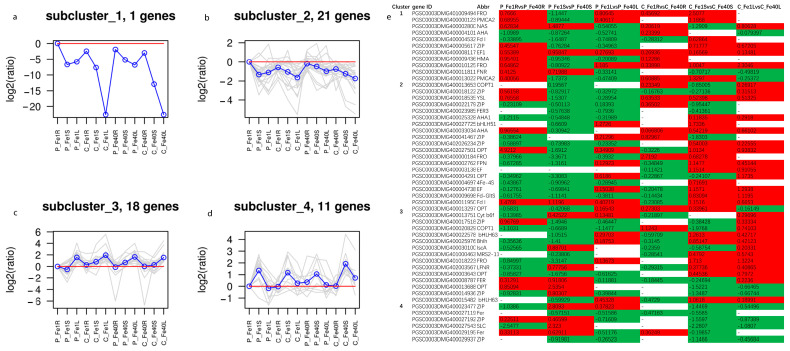
Expression patterns (**a**–**d**) and log2FoldChange (**e**) of 51 DEGs related to metal transport in different tissues of 05P and CI5 under Fe-deficient and control conditions. Gene expression patterns were determined by K-means clustering based on the log2FoldChange of FPKM + 1 relative to P_Fe1R. Red and blue lines represent the baseline and final cluster centers, respectively. Red and green highlights indicate upregulated and downregulated DEGs, respectively. Sample information is detailed in Table 1.

**Figure 17:**
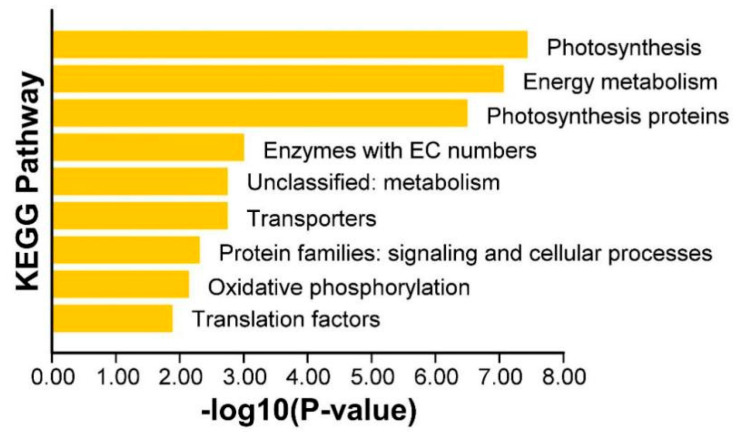
KEGG pathway enrichment analysis of 51 DEGs differentially expressed in at least four out of six pairwise comparisons.

To further investigate the expression patterns of iron-related genes, 51 genes that were differentially expressed in at least four of the six comparison groups (P_Fe1RvsP_Fe40R, P_Fe1SvsP_Fe40S, P_Fe1LvsP_Fe40L_ALL, C_Fe1RvsC_Fe40R, C_Fe1SvsC_Fe40S, and C_Fe1LvsC_Fe40L) were selected for hierarchical clustering and K-means cluster analysis (Supplementary S2). Hierarchical clustering grouped these genes into six clusters: Cluster 1, Cluster 4, and Cluster 6 primarily contained 10, 5, and 1 genes, respectively, with low expression levels; whereas Cluster 2, Cluster 3, and Cluster 5 contained 13, 15, and 7 genes, respectively, mainly with high expression levels (Figure 15). According to K-means cluster analysis, the 51 genes were divided into four expression subgroups (Figure 16a–d). Subcluster 1 contained only one *FRO* gene, which was upregulated in CI5 and 05P roots under iron deficiency stress and was expressed at a higher level in 05P roots (Figure 16a). Subcluster2 consisted of 21 DEGs with relatively low expression levels, which were expressed at higher levels in roots under iron deficiency stress (Figure 16b). Subclusters 3 and 4 contained 18 and 11 DEGs, respectively; DEGs in Subcluster 3 showed higher expression in leaves, while those in Subcluster 4 were more highly expressed in stems (Figure 16c,d). Additionally, KEGG pathway enrichment analysis of the selected 51 DEGs revealed significant enrichment in the Photosynthesis, Energy metabolism, and Photosynthesis protein pathways (Figure 17).

### 3.9. Real-Time Fluorescence Quantitative PCR Verification of DEGs

**Figure 18:**
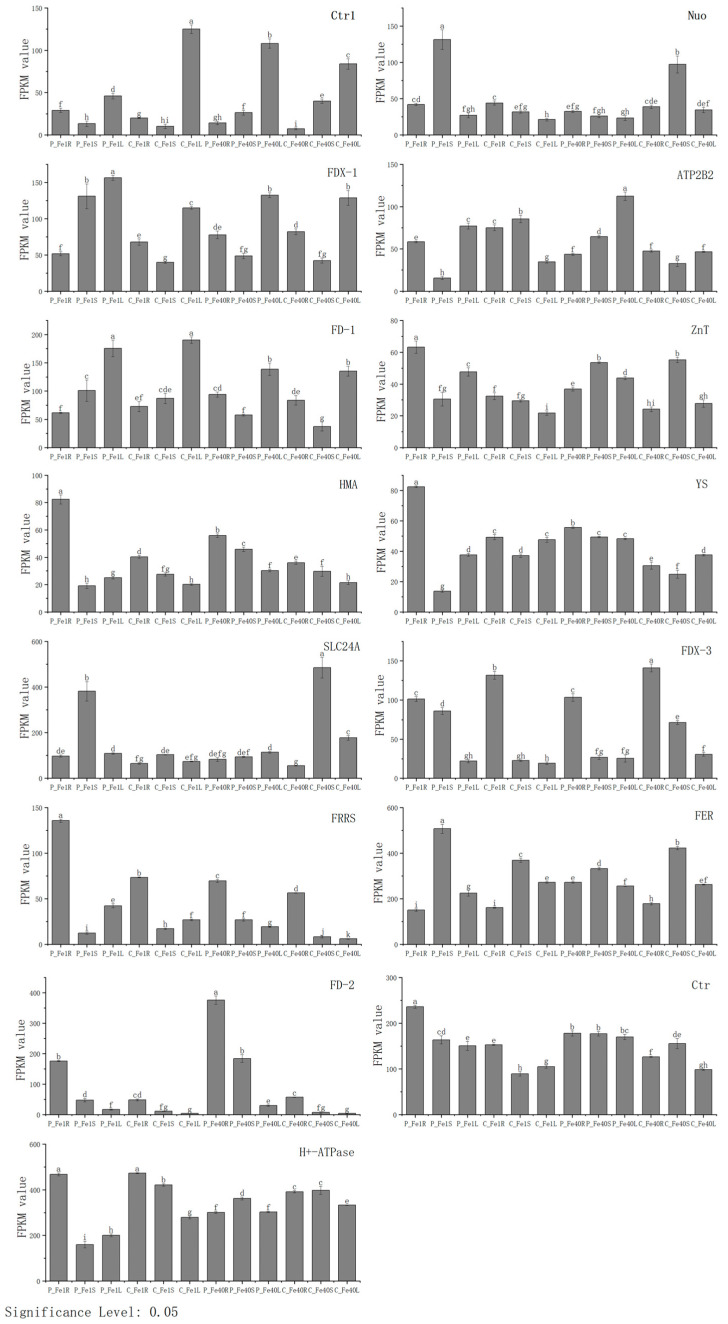
FPKM values of 15 differentially expressed genes in the roots and leaves of 05P and CI5 under iron deficiency stress. Different letters above error bars indicate values are significantly different at the 0.05 level according to Fisher’s LSD test. Sample information is detailed in Table 1.

**Figure 19:**
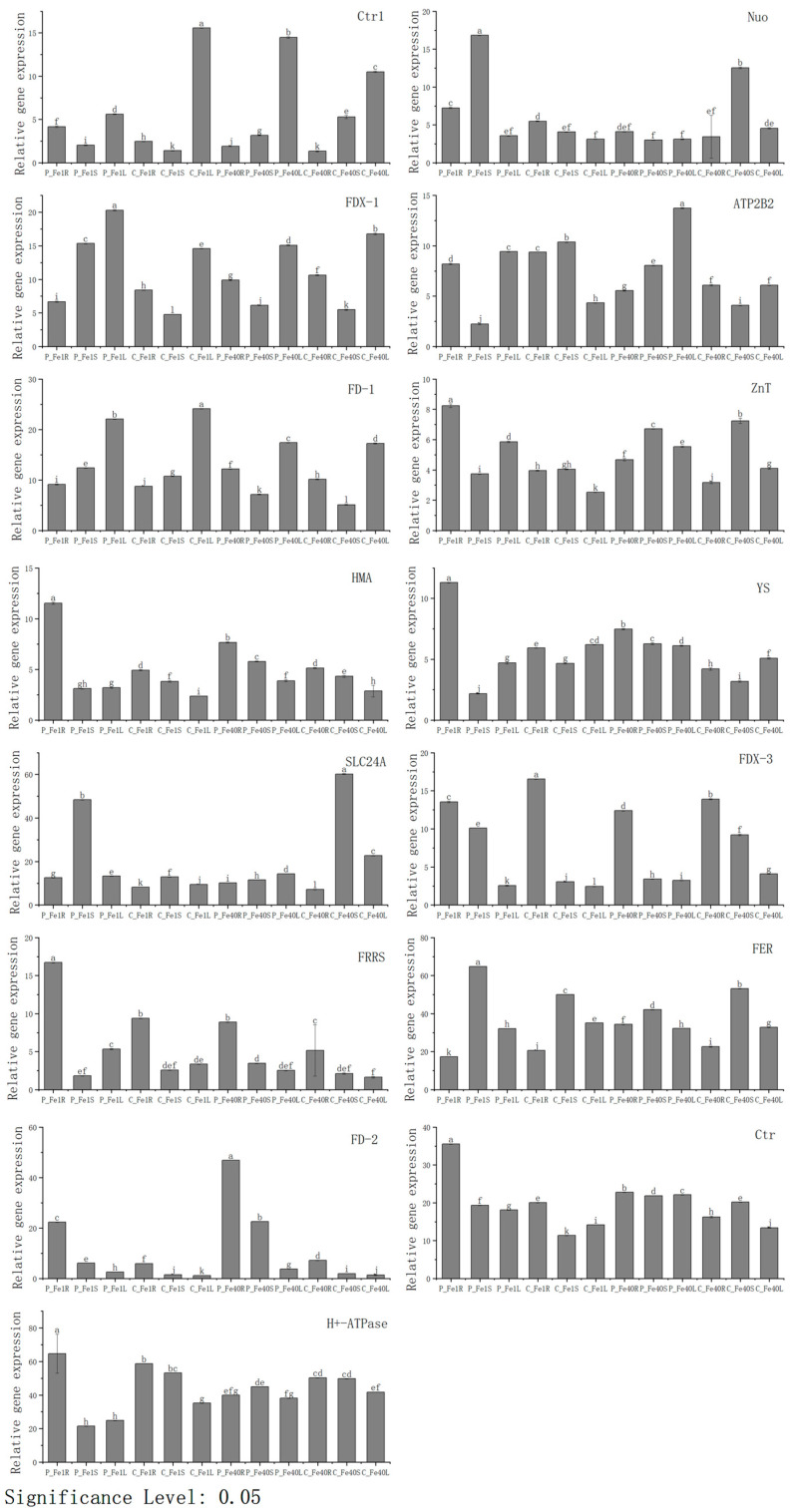
Relative expression levels of 15 genes in the roots and leaves of 05P and CI5 under iron deficiency stress (qRT-PCR). Different letters above error bars indicate values are significantly different at the 0.05 level according to Fisher’s LSD test. Sample information is detailed in Table 1.

To further validate the accuracy of the transcriptome sequencing data, qRT-PCR experiments were performed. Fifteen differentially expressed genes (DEGs) involved in iron absorption and transport were selected from the key regulatory networks identified through GO and KEGG enrichment analysis. The FPKM values of these 15 DEGs from the transcriptome sequencing data are shown in Figure 18, while their relative expression levels determined by qRT-PCR are presented in Figure 19. As illustrated in Figure 18 and Figure 19, the expression trends of these genes were highly consistent between the transcriptome sequencing and qRT-PCR results, confirming the reliability of the transcriptomic data. Overall, under iron deficiency stress, the expression patterns of these genes varied not only across different tissues of the same cultivar but also in the same tissues of different cultivars.

## 4. Discussion

Research on the mechanisms of plant responses to iron deficiency has primarily focused on the model species Arabidopsis thaliana. Related studies have also been conducted in crops susceptible to iron deficiency chlorosis, such as soybean, peanut, and apple [38,39,40]. As a globally important staple crop, the mechanisms underlying potato (*Solanum tuberosum* L.) responses to iron deficiency remain less characterized compared to model plants like Arabidopsis and major crops such as soybean. This study provides a systematic investigation of physiological and transcriptomic adaptations to iron deficiency in potato, revealing cultivar-specific strategies that advance our understanding of iron homeostasis regulation in non-model crops.

Iron deficiency primarily affects chlorophyll synthesis and photosynthetic system functionality in potato plants. Our study revealed a significant reduction in leaf SPAD values in both potato cultivars (Figure 10), with the low-iron cultivar CI5 showing a more pronounced decrease, consistent with findings in lettuce by Roosta et al. [41]. The decline in chlorophyll content directly leads to interveinal chlorosis (Figure 1), a phenomenon initially reported by Abadía et al. [42]. The underlying mechanism likely involves impaired function of PSII and PSI, particularly damage to the PSII reaction center [43,44,45]. As an essential component of Fe-S clusters, heme, and other key elements in photosynthetic electron transport [41,46], iron deficiency reduces photochemical efficiency and destabilizes thylakoid membranes [47,48], ultimately inhibiting plant growth, as evidenced by decreased plant height and fresh shoot weight (Figure 2 and Figure 4), similar to observations in iron-deficient soybean [49].

Iron deficiency triggers reactive oxygen species (ROS) accumulation, as indicated by the progressive increase in malondialdehyde (MDA) content (Figure 9), analogous to findings in salt-stressed wheat [50]. To counteract oxidative stress, potato plants activate their antioxidant system, leading to elevated activities of superoxide dismutase (SOD) and peroxidase (POD) (Figure 7 and Figure 8), mirroring responses observed under drought stress [51,52]. Additionally, osmoprotectants such as soluble sugars and proteins accumulate to maintain cellular osmotic balance, enhancing stress tolerance—a strategy also documented in drought-resistant potato genotypes [53,54,55].

Iron deficiency significantly enhances the absorption and accumulation of divalent cations, including Zn, Mg, Ca, and Mn (Figure 12), corroborating reports in peanut [56], Arabidopsis [57,58,59,60], and other species. This phenomenon may be attributed to the broad substrate specificity of iron transporters, such as Arabidopsis IRT1, which facilitates the uptake of Zn^2+^, Mn^2+^, and other metals [61,62]. Transcriptomic analysis identified the upregulation of Iron-regulated transporter genes specifically in roots (Supplementary S1), suggesting that potato modulates metal transport systems in response to iron deprivation.

GO and KEGG enrichment analyses elucidated the molecular basis of iron uptake in potato:

1. Rhizosphere acidification: Two H^+^-ATPase genes (H(+)-transporting ATPase, *PGSC0003DMG400004101*; Plasma membrane H^+^-ATPase, *PGSC0003DMG400033034*) exhibited differential expression, functionally resembling *AHA2* in Arabidopsis [63] and *MxHA7* in Malus xiaojinensis [64].
2. Fe^3+^ reduction: Four *FRO-like* genes (e.g., *PGSC0003DMG400000184*) were upregulated in roots, consistent with the pivotal role of *FRO2* in Arabidopsis [63,65].
3. Fe^2+^ transport: Two *IRT* homologs (*Iron-regulated transporter 1/2*, *PGSC0003DMG400010343/369*) were specifically induced in roots, analogous to *AtIRT1* [66].

Furthermore, the enrichment of Zinc ion binding protein and Ferritin genes in photosynthesis and energy metabolism pathways implies their potential involvement in iron homeostasis, warranting further investigation.

## 5. Conclusions

This study systematically elucidates the comprehensive effects of iron deficiency stress on the physiological, biochemical, and molecular regulatory networks in potato, with the main innovations highlighted in the following three aspects:

(1) First elucidation of the physiological compensation mechanism via ionomic remodeling under iron deficiency
  The study reveals that iron deficiency not only induces typical chlorosis symptoms (SPAD value reduced by 42.3%) and growth inhibition (biomass decreased by 35.7%) but also triggers a unique ionomic reprogramming, characterized by the specific accumulation of divalent metals such as Zn, Mg, Ca, Mn, and Cu in various tissues (increased by 20–150%). This multi-element synergistic regulation suggests that potato may activate a metal compensation mechanism through ionomic remodeling, providing new insights into plant micronutrient homeostasis.
(2) Deciphering the molecular switch network of iron uptake and transport in potato
  Through whole-genome transcriptome analysis, we constructed, for the first time, a three-tiered iron absorption regulatory module (“H^+^-ATPase–FRO–IRT”) in potato roots. Under iron deficiency, root-specific induction of H^+^-ATPase genes (e.g., *PGSC0003DMG400004101*, up-regulated 5.2-fold) promotes rhizosphere acidification, followed by activation of the iron reduction system mediated by *FRO* family genes (*PGSC0003DMG400000184* et al., up-regulated 3.8–7.1-fold), and finally facilitates Fe^2+^ transmembrane transport via *IRT* transporters. This regulatory cascade provides novel targets for genetic improvement of iron nutrition in crops.
(3) Uncovering the synergistic defense strategy of oxidative stress and carbon metabolic reprogramming
  This study provides the first evidence that iron deficiency triggers a dual defense response in potato: (i) activation of the SOD/POD antioxidant system (activity increased by 2.3–4.5-fold) to alleviate membrane lipid peroxidation (MDA accumulation elevated by 89%), and (ii) remodeling of carbon metabolism, manifested by a significant increase in soluble sugars and proteins (by 60–80%). This metabolic reprogramming may supply carbon skeletons for iron chelate synthesis, offering new perspectives for understanding plant adaptation mechanisms under iron stress.

These findings not only advance the theoretical framework of iron nutrition in tuber crops but also provide molecular targets and a scientific basis for developing iron-deficiency-resistant potato cultivation strategies.

## Figures and Tables

**Table 1:** Sample information.

Variety	Sampling Tissue	Iron Deficiency Stress (FeNaEDTA: 1 mg/L)	Control (FeNaEDTA: 40.4 mg/L)
		Sample Naming	Sample Naming
05P	root	PFe1R	PFe40R
	stem	PFe1S	PFe40S
	leaf	PFe1L	PFe40L
CI5	root	CFe1R	CFe40R
	stem	CFe1S	CFe40S
	leaf	CFe1L	CFe40L

**Table 2:** Primer Information.

Gene ID	Gene Name	Forward Primer (5′-3′)	Reverse Primer (5′-3′)
PGSC0003DMG400020829	*Ctr1*	GCATGGAATGGCTATGGGGC	ATCATCATCCCGCTACCCGC
PGSC0003DMG400028681	*Nuo*	TACAGGGAACAAAGGGCGAT	ATTCGGTTCCTACGTTGATACTCT
PGSC0003DMG400035711	*FDX-1*	TGCAAGCTAGGAGTGTGCAT	CAATCATCGGCAGTTGCGAG
PGSC0003DMG400013022	*ATP2B2*	AGGAGCCTTTGTGGTGTTGT	CACTTGGTTGCATGCCTTGTAT
PGSC0003DMG400011950	*FD-1*	CCGAGGAAACCGGGATAGAT	AACGCAAGTGAGCACAAATCC
PGSC0003DMG400022179	*ZnT*	CAAGGGCAGTCACAGGTCT	TGGCTATTCCCTCAAAGACGG
PGSC0003DMG400009436	*HMA*	TGCAGTTGCTAAGGAGGTTGG	AACGGACGACCTCAGCTTTC
PGSC0003DMG400018535	*YS*	AGTATTGCTGTAGGAGGTGGC	TGCCACTTGTATCAACCCCAG
PGSC0003DMG400027543	*SLC24A*	TCTGCTCCTTATTTCCTCCGTT	CAGTGGCACACCATCCTGAG
PGSC0003DMG400026360	*FDX-3*	TGCTGCCAACTTTGCCTAAC	ACGGTCGACATGTTTTACCAAT
PGSC0003DMG400010125	*FRRS*	GAAAGGGAGCTGGAAAGCCT	TGGTAGAAGCAAGCAGAGAGC
PGSC0003DMG400008787	*FER*	AGTGCAGAGGAAAGAGAGCAC	CGCAAGCTCCATTGCATAC
PGSC0003DMG400004532	*FD-2*	TGCGGACACGTGGAAGATTA	TGTGCTTGGCTGGATCTCAC
PGSC0003DMG400013653	*Ctr*	TACGGCGATCGTTTTTGGGA	TGACATCATCCTCATCGCCG
PGSC0003DMG400004101	*H^+^-ATPase*	GCTGGAGTATTTCCTGAGCAC	AAGCAGGAGCATCGTTCACA
Reference Genes	*StActin*	AGATGCTTACGCTGGATGGAATGC	TTCCGGTGTGGTTGGATTCTGTTC

## Data Availability

All data supporting the findings of this study are available within the paper and within its Supplementary Data published online.

## Supplementary Materials

The following supporting information can be downloaded at: https://www.mdpi.com/article/10.3390/plants14182934/s1, Supplementary S1: (FPKM and log2FoldChange of the 137 DEGs related to metal in the tissues of 05P and CI5 under Fe-deficient conditions). Supplementary S2: FPKM and log2FoldChange of the 51 DEGs at least differentially expressed in four out of six pairs for 05P and CI5 under Fe-deficient and control conditions.
